# Human Milk Oligosaccharides and Infant Neurodevelopment: A Narrative Review

**DOI:** 10.3390/nu15030719

**Published:** 2023-01-31

**Authors:** Paige K. Berger, Margaret L. Ong, Lars Bode, Mandy B. Belfort

**Affiliations:** 1 Department of Pediatric Newborn Medicine, Brigham and Women’s Hospital, Harvard Medical School, Boston, MA 02115, USA; 2 Department of Pediatrics, Mother-Milk-Infant Center of Research Excellence (MOMI CORE), Human Milk Institute (HMI), University of California, La Jolla, CA 92093, USA

**Keywords:** infant, breastfeeding, human milk, oligosaccharides, neurodevelopment, cognitive development

## Abstract

The objective of this narrative review was to synthesize the literature on human milk oligosaccharides (HMOs) and neurodevelopmental outcomes in human milk-fed infants. We conducted a scoping review of the literature indexed in PubMed reporting observational or interventional studies on HMO exposure in relation to psychometric measures in infants. Studies were characterized based on study design and definitions of HMO exposure and neurodevelopmental outcomes. Six studies were identified; all were observational in design, and five were conducted in full-term infants. Sample sizes ranged from 35–659 infants. HMOs were defined as individual concentrations or relative abundances assessed at 1 and/or 6 months of age. Studies accounted for differences in HMO exposure based on maternal secretor status. Neurodevelopmental outcomes were assessed between 6 and 24 months of age and included four domains. Studies in full-term infants reported that total and individual fucosylated and sialylated HMOs were positively associated with cognitive, language, and motor skill domains between 18 and 24 months of age, while the single study in preterm infants reported no statistically significant findings in the full cohort. The presence of a maternal secretor did not consistently alter the associations between HMO exposure and neurodevelopmental outcomes. Emerging evidence from observational studies suggests that HMO exposure may be beneficial for neurodevelopment in infants.

## 1. Introduction

Fetal life and infancy are the most rapid periods of postnatal brain growth, which provides the structural support for neurodevelopmental outcomes into childhood [1,2]. Because neurodevelopmental outcomes influence higher-order functions into adulthood [3,4], it is critical to comprehend the early influences of brain maturation that can be altered to improve cognitive, language, motor skill, and social-emotional capacities in the long run. Identifying modifiable determinants of neurodevelopmental outcomes is important for all infants and is particularly pressing for preterm infants, who are at elevated risk for neurodevelopmental deficits due to prematurity-related brain injury, inflammation, and undernutrition in the neonatal intensive care unit (NICU) [5,6,7,8].

Among full-term and preterm infants, human milk is the optimal source of nutrition for many reasons, including for brain development and neurodevelopmental outcomes [9,10,11]. Leveraging the beneficial components of human milk for preventive and/or therapeutic interventions holds promise, including for interventions specific to the NICU environment. The benefits of breastfeeding and human milk exposure are well-documented, with studies reporting positive associations between the duration of human milk exposure and total and region-specific brain volumes in infancy and intelligence test scores in early childhood and beyond for those born full-term and preterm [11,12,13,14]. Although the macronutrient content of human milk has been examined in relation to brain development and neurodevelopmental outcomes [15,16], the composition of human milk is one of the most understudied biological systems in the life sciences. There is also a knowledge gap regarding the mechanisms through which human milk components shape the structural and functional features of the infant brain.

In recent years, studies have started to shift focus from the nutrient composition of human milk to non-nutrient components known as “human milk bioactives”. Many human milk bioactives are molecules that protect the nursing infant against infection and inflammation and promote the organization of microbial communities to support the maturation of organs and systems, a process known as lactocrine programming [17,18]. Although human milk contains hundreds to thousands of distinct bioactives, human milk oligosaccharides (HMOs), the third most abundant solid component of human milk after lactose and lipids, are promising targets to better understand the role of breastfeeding on brain development via lactocrine programming. HMOs are structurally diverse complex carbohydrates that include more than 150 distinct structural permutations via HMO biosynthesis, which follows a basic blueprint beginning with a lactose molecule that is elongated and/or fucosylated or sialylated to create various subgroups [19]. Slight differences in HMO structure confer diverse physiological functions that can influence brain maturation and neurodevelopment [20]. HMOs are a source of prebiotics for the developing gut microbiome, which can prevent inflammation and lead to the production of metabolites with far-reaching effects on the brain as part of the gut-brain axis [21]. In addition, HMOs have microbiome-independent effects in the gut and beyond, which may also alter brain structure and function [17]. Moreover, HMOs may also be a direct or indirect source of sialic acid, an essential nutrient for the organization of brain tissues [22,23].

Animal studies have identified specific HMOs that may influence early brain maturation [22,24,25]. Human studies have started to examine these candidate HMOs in relation to neurodevelopmental outcomes, although there has been more focus on associations in full-term infants as opposed to preterm infants [26,27,28,29,30,31]. It is critical to describe the current state of knowledge on HMO exposure and neurodevelopmental outcomes in all human milk-fed infants, which may have clinical relevance, particularly for preterm infants, and/or help identify knowledge gaps that need to be addressed in future studies. The purpose of this narrative review was to identify and synthesize studies on associations of HMO exposure during infancy with neurodevelopmental outcomes through 24 months of age and discuss the potential nutritional implications, including those for preterm infants.

## 2. Materials and Methods

In October 2022, we conducted a literature search on PubMed using the terms “infant”, “human milk”, “oligosaccharides”, and “cognitive development”, which produced 29 results. We used article titles and abstracts to identify studies that met these criteria: (1) study design was observational or interventional; (2) cohort included human milk-fed infants born full-term (≥37 weeks) and/or preterm (<37 weeks); (3) methods defined HMO exposure and described analysis; (4) methods defined neurodevelopmental outcomes and described psychometric assessments; and (5) psychometric assessments were conducted within 24 months of age. To ensure that our search was complete, we also reviewed articles within our own collections and related bibliographies using the “see all similar articles” feature in PubMed for the previously identified articles. For each result that met our criteria, we extracted information including the author, sample size, definition of exposure, definition of outcome, covariates, and key findings.

## 3. Results

We identified six studies that met our inclusion criteria, all of which were observational in design [26,27,28,29,30,31]. Of these studies, five were conducted in full-term infants, and one was conducted in preterm infants. Table 1 presents the extracted information. Sample sizes ranged from 35 to 659 participants. Overall, exposure to HMOs was defined as individual concentrations (e.g., μg/mL or mg/L) or relative abundances (e.g., % of total measured HMOs) in milk assessed at 1 month and/or 6 months of age. All studies examined at least one of the following HMOs, which together account for >90% of total HMO composition: 2′-fucosyllactose (2′FL), 3-fucosyllactose (3FL), 3′-sialyllactose (3′SL), 6′-sialyllactose (6′SL), difucosyllactose (DFLac), lacto-N-tetraose (LNT), lacto-N-neotetraose (LNnT), lacto-N-fucopentaose (LNFP) I, LNFPII, LNFPIII, sialyl-LNT (LST) b, LSTc, difucosyl-LNT (DFLNT), disialyl-LNT (DSLNT), lacto-N-hexaose (LNH), fucosyl-LNH (FLNH), difucosyl-LNH (DFLNH), fucosyl-disialyl-LNH (FDSLNH), and disialyl-LNH (DSLNH). Studies also accounted for differences in concentrations of HMOs based on maternal genetics, namely secretor status. For reference, mothers classified as secretors have an active secretor locus (Se+) that encodes for a functional fucosyltransferase-2 enzyme. Secretors produce higher concentrations of distinct HMOs (i.e., alpha-1-2-fucosylated HMOs, primarily 2′FL) than non-secretors (Se−).

Neurodevelopmental outcomes were assessed between 6 and 24 months of age and included the following domains: (1) cognitive; (2) language; (3) fine and gross motor skills; and (4) social-emotional. These domains were measured through various psychometric assessments, including the Bayley Scales of Infant and Child Development (BSID-III), the Kilifi Developmental Inventory (KDI), the MacArthur-Bates Communicative Development Inventories (MB-CDIs), the Mullen Scales of Early Learning (MSEL), and the Ages and Stages Questionnaire (ASQ). The combination of covariates included in statistical models varied between studies, although half of them adjusted for maternal age, pre-pregnancy weight (BMI), and education level collected at baseline.

Overall, studies found significant associations between exposure to HMOs during breastfeeding and neurodevelopmental outcomes in infants. Specifically, most studies reported that exposure to specific and total fucosylated and sialylated HMOs during early lactation (i.e., 1 month) was associated positively with measures of cognitive, language, and motor skill development in later infancy (i.e., 18 to 24 months). For example, Berger et al. reported that infants exposed to higher concentrations of the most abundant fucosylated HMO, 2′FL, at 1 month but not 6 months had higher cognitive development scores using the BSID-III at 24 months of age. For every 1 standard deviation increase in 2′FL concentration, there was a 0.59 standard deviation increase in the cognitive development score [26]. Similarly, Oliveros et al. reported that exposure to 2′FL at 1 month associated positively with overall motor skill development scores, a composite of fine and gross development subdomain scores, using the BSID-III at 6 months of age (β = 0.003, *p* = 0.04), while exposure to 6′SL, the most abundant sialylated HMO, at 1 month associated positively with overall motor skill (β = 0.02) and cognitive development scores (β = 0.02) at 18 months of age (*p* ≤ 0.04) [29]. After adjusting for maternal education level, gestational weight gain, and paternal IQ, however, there were no significant associations between 2′FL exposure and neurodevelopmental outcomes.

In line with the above findings, Cho et al. reported that infants exposed to higher concentrations of the sialylated HMO 3′SL, the structural isomer of 6′SL, had higher early learning composite scores using the MSEL at a mean age of 10 months [*p*-value, effect size (EF), 95% CI: *p* < 0.01, EF = 13.1, 95% CI = 5.36–20.8], attributed to higher expressive (*p* = 0.02, EF = 9.95, 95% CI = 3.91–16.0) and receptive (*p* = 0.05, EF = 7.53, 95% CI = 2.51–13.8) language subdomain scores among older infants (i.e., >12 months) [28]. These findings, however, were specific to infants of mothers with blood type A (A-tetra+) and mothers classified as secretors (Se+) [28]. Similarly, Jorgensen et al. reported that exposure to total fucosylated (*p* < 0.01) and total sialylated HMOs (*p* = 0.03) at 6 months was associated positively with language scores at 18 months of age among infants of secretor mothers but not in infants of non-secretor mothers [27]. There were additional significant associations of specific HMO concentrations with neurodevelopmental outcomes that were also distinct based on maternal secretor status (Table 1).

Rozé et al. conducted the first (and only) study in preterm infants, which found that exposure to specific and total HMOs in the first 7 weeks did not associate significantly with neurodevelopmental outcomes at 24 months of age in the full cohort of preterm infants [31]. In a subgroup analysis that included preterm infants of secretor mothers only, exposure to the fucosylated HMO, LNFPIII, was associated positively with the total ASQ score (standardized beta, 95% CI, *p*-value: β = 0.11, 95% CI = 0.03–0.19, *p* = 0.01), a composite of communication, gross motor, fine motor, problem solving, and social-emotional skill scores [31]. Taken together, findings in infants born full-term and preterm underscore the influence of maternal blood groups and genetics on the production of distinct HMOs, which could theoretically affect associations with neurodevelopmental outcomes over 24 months of age [27,28,31].

## 4. Discussion

The studies identified in this narrative review largely lend support to the influence of HMOs on neurodevelopmental outcomes in human milk-fed infants born full-term and preterm. These findings are an important step in characterizing the components of human milk that are most beneficial for brain development, with implications for future behavior, academic performance, and vocational training. The literature also highlights the role of “lactotypes”, or maternal blood groups, and secretor status, a determinant of HMO production and compositional profile [32,33], which influence the “dose” or concentration of HMOs to which infants are exposed. It remains unclear from these studies, however, whether differences in “lactotypes” would affect the compositional profile of HMOs to such an extent that it would create differences in brain maturation and neurodevelopment in infants based on maternal blood groups or secretor status.

Overall, findings revealed that greater concentrations of HMOs in human milk during the recommended window of exclusive breastfeeding, a period of dramatic growth and experiential learning, were associated with greater cognitive, language, and motor skill development later in infancy (i.e., 18 to 24 months of age). The studies generally found that the same individual fucosylated and sialylated HMOs were significantly associated with neurodevelopmental outcomes in human milk-fed full-term infants. Specifically, 2′FL, 3′SL, and 6′SL emerged as promising candidates and prime initial targets to better understand the role of HMOs and breastfeeding on brain maturation, an important observation given that several of the studies examined additional HMOs of the more than 150 distinct structural permutations of HMOs identified to date and reported inconsistent or null findings in relation to neurodevelopmental outcomes.

Another similarity among the studies was that the influence of maternal blood groups and secretor status on the association of HMO exposure with neurodevelopmental outcomes was highlighted and accounted for. While one study adjusted for maternal secretor status [26], several studies separated their samples into maternal secretors and non-secretors [27,28,30,31]. This method may be justified, as maternal secretor status is an established influence on HMO profile and could therefore be a potential effect modifier of associations with neurodevelopmental outcomes: concentrations of almost all HMOs differ between maternal secretors and non-secretors [32], which could conceivably influence the “dose” of HMOs delivered to human milk-fed infants, thereby contributing to disparities in neurodevelopment. Three studies reported that total and individual fucosylated and sialylated HMOs associated positively with early learning and language development scores in infants of secretor mothers only [27,28,31], while another found no significant associations when the sample was separated by secretor status [29]. Studies with larger and more balanced sample sizes among the main secretor and Lewis blood groups are needed to understand variations in HMO exposures that could conceivably contribute to incongruences in the neurodevelopmental outcomes of infants [28]. Findings from these studies would also lend insight into the potential risks and benefits of exposure to secretor and non-secretor milk, which may dictate whether screening human milk for secretor status is warranted in clinical settings and whether HMO supplementation strategies are justified. Further, none of the studies included information on the Secretor and Lewis genotypes of the receiving infant, which define the endogenous glycan environment in the infant gut and would provide valuable information on the role of genetic patterns. Together with exogenous glycans provided as HMOs from human milk, these glycans may be important for shaping microbial communities in the infant gut and differentially influencing the gut-brain axis. Both maternal and infant Secretor and Lewis genotypes should be included in future studies to better capture the mother-milk-infant “triad”, in which each part of the triad (mother, milk, and infant) has their own microbiome(s) that are connected starting at birth and through lactation [34].

Findings in infants are in line with what has been reported in animal studies, which have also shown that 2′FL, 3′SL, and 6′SL enhance neurodevelopment through diverse physiological functions in brain maturation. For example, rat and mouse pups fed 2′FL, 3′SL, and 6′SL performed better on cognitive tasks compared to controls due to enhanced long-term potentiation (LTP) in the hippocampus and prefrontal cortex [22,24,35], which reflects strengthening of synaptic connections that foster learning and memory [36,37]. HMOs may enhance LTP in the brain through their role as prebiotics in the gut, where HMOs undergo bacterial fermentation to produce metabolites that cross the blood-brain barrier. In vitro, these metabolites (e.g., short-chain fatty acids) enter the central nervous system as energy for cellular metabolism and/or induce the expression of proteins that strengthen synaptic connections and LTP, including brain-derived neurotrophic factor and phosphorylated calcium/calmodulin-dependent kinase II [38,39,40]. 3′SL and 6′SL also contain sialic acid, an essential nutrient for ganglioside formation and myelination that increase information processing speed in the brain [25,41,42]. Animal and cell culture studies therefore provide biological bases for the observed associations of individual fucosylated and sialylated HMOs with neurodevelopment in infants.

All but one of the studies identified in this narrative review were conducted in full-term infants. Indeed, findings from this single study lend support to the premise that HMO exposure may have unique neurodevelopmental benefits for preterm infants [31]. Preterm infants are more susceptible to neurodevelopmental deficits into childhood, which result from brain injuries occurring around the time of birth as well as impaired brain maturation while in the neonatal intensive care unit (NICU). Preterm infants who are fed human milk, however, have improved neurodevelopmental outcomes in the NICU, particularly preterm infants born <30 weeks of gestation [43]. Moreover, quantitative MRI studies of the preterm brain demonstrate that human milk feeding is associated with more mature cerebral white matter, less injury, and larger regional volumes, providing potential pathways to better neurodevelopmental outcomes in this population [10,44,45]. There remains, however, a gap in our understanding of which components of human milk may be responsible for neuroprotective effects. Although Rozé and colleagues identified exposure to LNFPIII as being relevant for neurodevelopment among preterm infants, there is a need for studies that examine associations of HMO exposure with brain growth, maturation and injury in the NICU [31]. These studies are indicated given findings in animal models, which have shown that fucosylated and sialylated HMOs support the formation of gangliosides and myelination [24,25], which may repair white matter injury and enhance connectivity in the preterm brain.

The studies highlighted in this narrative review have several limitations. All studies were observational in design. Although most maternal characteristics are measured and adjusted for because they may be shared determinants of HMO composition in milk and infant neurodevelopmental outcomes [46], residual confounding is possible. Further, different studies adjusted for different sets of covariates and/or did not include all potential confounders and effect modifiers. For example, studies in full-term infants did not report antibiotic exposure, which could theoretically impact the gut microbiome and the proposed mechanism through which HMOs may influence neurodevelopmental outcomes. While most studies adjusted for multiple comparisons, several did not, which is important given that the number of HMOs and hypotheses that were tested increases the odds of committing a Type 1 error. Another limitation was that several studies included infants who received formula in addition to human milk but did not report the volume of formula or human milk intake. It is plausible that the volume of formula or human milk consumed by the infant would impact the dose of HMOs, thereby affecting neurodevelopmental outcomes. Future studies should report the volume of formula and human milk received by the infant and include it in statistical analyses as a potential effect modifier or covariate. Moreover, the studies differed in the psychometric assessment tools used to calculate cognitive, language, and motor skill development scores, which makes it difficult to compare results. Despite these differences, however, all studies reported similar findings.

## 5. Conclusions

In this narrative review, we present observational evidence suggesting that HMO exposure during early breastfeeding may enhance neurodevelopmental outcomes in later infancy. The benefits of breastfeeding on brain development are well-documented, but the exact constituents of milk that may underlie favorable associations have not been established. The human studies conducted to date indicate that fucosylated and sialylated HMOs are viable candidates, reporting positive associations with cognitive, language, and motor skill development domains in human milk-fed infants [26,27,28,29,31]. These observational data, taken together with insights about mechanisms from animal and cell culture studies, provide rationale for examining associations of HMO exposure with robust imaging measures of brain development in full-term infants and brain injury in larger and more balanced cohorts of preterm infants, as well as psychometric assessments in follow-up studies. Moreover, future clinical trials are warranted to validate the roles of HMOs on brain development and cognitive functioning, which may guide new interventions and support recommendations for nutritional care of preterm infants in the NICU.

## Figures and Tables

**Table 1:** Summary of Observational Studies on Associations of HMOs with Infant Neurodevelopmental Outcomes ^1^.

Author and Year	Sample Size	Exposure Definition	Outcome Definition	Model Covariates	Key Findings
Berger 2020 [26]	50 Exclusively breastfed	**At 1 and 6 months:** Concentrations (μg/mL) of 19 HMOs, initial interest in 2′FL	**At 24 months:** Cognitive scores via BSID-III	**At 1 month:** Secretor status Age at delivery Maternal education level Infant sex Infant age Infant birthweight Did not correct for multiple comparisons	**Total sample:** 2′FL at 1-month, but not 6 months, associated positively with cognitive scores at 24 months (standardized beta, *p*-value: β = 0.59, *p* < 0.01) DSLNT at 1 month, but not 6 months, associated inversely with cognitive scores at 24 months (β = −0.32, *p* < 0.02) There were no significant associations between the remaining HMOs and cognitive scores at 24 months
Jorgensen 2020 [27]	659 Breastfeeding exclusivity not reported.	**At 6 months:** Abundance (%) of total, Fuc, Sia, and non-Fuc neutral HMOs Relative abundance (%) of 51 HMOs	**At 12 months:** Standing Walking **At 18-months:** Motor skill scores via KDI Language scores via MB-CDIs Social-emotional via PSED Working memory and executive function via an A-not-B task	**At 6 months:** Maternal age Maternal height Maternal BMI Parity Maternal education level Food security Maternal HIV status Maternal hemoglobin Household assets Location Infant sex Season Study group (parent study) Family Care Indicator Score (for an 18-month outcome) Child mood, activity level, and cooperation (motor/executive function models only) Corrected for multiple comparisons	**Total sample:** Mothers with total Fuc HMOs above vs. below the median had infants with greater vocabulary [median (95% CI): 22.7 (12.9–35.1) words vs. 18.2 (9.6–29.4) words; *p* = 0.03] Mothers with total Sia HMOs above vs. below the median had infants with greater vocabulary [median (95% CI): 23.9 (13.6–37.0) words vs. 19.3 (10.5–30.7) words; *p* = 0.03] **Secretors only:** Total Fuc (*p* < 0.01) and total Sia HMOs (*p* = 0.03) associated positively with language scores LNnT and LNH are associated inversely with language scores (Ps < 0.01) LSTc was associated inversely with socio-emotional scores at 18 months (*p* = 0.01) DFLNH associated positively with working memory and executive function (*p* = 0.02) 6′SL associated inversely with walking (*p* < 0.01) **Non-secretors only:** LNFPII associated positively with motor skill scores (*p* = 0.02) LSTb associated positively with working memory and executive function (*p* < 0.01) DFLNH was associated inversely with socio-emotional (*p* = 0.02) and positively with motor skill scores (*p* = 0.03)
Oliveros 2021 [29]	82 Breastfeeding exclusivity not reported	**At 1 month:** Concentrations (mg/L) of individual HMOs, 2′FL, and 6′SL	**At 6 and 18 months:** Cognitive scores Language scores Motor skill scores Social-emotional scores All assessments are performed via BSID-III	**At 1 month:** **Model 1** included gestational weight gain, maternal education level, and paternal IQ **Model 2** included only the pre-pregnancy weight and parent study group assignment Did not correct for multiple comparisons	**Total sample, Model 1:** There were no significant associations of 2′FL with neurodevelopmental outcomes 6′SL associated positively with cognitive scores at 18 months (standardized beta, *p*-value: β = 0.05, *p* = 0.04) **Total sample, Model 2:** 2′FL (β = 0.003, *p* = 0.04) associated positively with motor skill scores at 6 months (*p* ≤ 0.04) 6′SL associated positively with motor skill (β = 0.02, *p* = 0.04) and cognitive scores (β = 0.02, *p* = 0.02) at 18 months There were no significant associations of 2′FL with neurodevelopmental outcomes in separate groups of secretors and non-secretors
Cho 2021 [28]	99 Exclusively breastfed (81%)	**At 2–25 months (mean, 10 months):** Concentrations (mg/L) of HMOs, 2′FL, 3FL, 3′SL, 6′SL, LNT, LNnT, LNFPI, A-tetra	**At 2–25 months** **(mean, 10 months):** Early learning composite scores Subdomain scores Fine motor Gross motor Visual reception Receptive language Expressive language All assessments were performed via MSEL	**At 2–25 months ** **(mean, 10 months):** Remaining HMOs analyzed but not included in the model Site and batch effects Corrected for multiple comparisons	**Total sample:** There were no significant associations of individual HMOs with MSEL outcomes when all samples analyzed together. **A-tetra+ only:** 3′SL associated positively with early learning composite scores [(*p* < 0.01; effect size (EF), 13.1; 95% CI, 5.36–20.80] 3′SL associated positively with receptive language scores (*p* = 0.05; EF, 7.53; 95% CI, 2.51–13.8) 3′SL associated positively with expressive language scores (*p* = 0.02; EF, 9.95; 95% CI, 3.91–16.0) **Secretors only:** 3′SL associated positively with early learning composite scores (*p* = 0.02) and receptive language scores (*p* = 0.045)
Ferreira 2021 [30]	35 Exclusively breastfed (67%)	**At 1-month:** Concentrations (nmol/mL) of 19 individual HMOs, Sia, Fuc	**At 1-, 6- & 12-months:** Communication Gross motor skills Fine motor skills Problem solving Personal-social skills All assessments performed via ASQ Inadequate development defined as ASQ sub-scores ≤ −2 SD from the mean	**At 1-month:** Gestational age at birth Gestational weight gain Pre-pregnancy BMI Maternal age Parity Mode of breastfeeding Corrected for multiple comparisons	**Total sample:** LNT associated inversely with risk of inadequate personal-social skill development scores [hazard ratio (HR), 0.06; 95% CI, 0.01–0.76)] LNT associated inversely with risk of having two or more inadequate ASQ scores (HR, 0.06; 95% CI, 0.01–0.59) **Secretors only:** LNT associated inversely with risk of inadequate personal-social skill development scores (HR, 0.09; 95% CI, 0.02–0.84) LNT associated inversely with risk of having two or more inadequate ASQ scores (HR, 0.05; 95% CI, 0.01–0.70)
Rozé 2022 [31]	137 Exclusively breastfed	**From 1 to 7 weeks** Concentrations (mg/L) of 19 HMOs, total HMOs, Sia	**At 24 months:** Communication Gross motor skills Fine motor skills Problem solving Personal-social skills All assessments were performed via ASQ	**At 1 week:** Gestational age at birth Birthweight z-score Infant sex Maternal education level Maternal income level Days on antibiotics Corrected for multiple comparisons	**Total sample:** There are no significant associations between individual and total HMOs and Sia with ASQ outcomes when all samples were analyzed together **Secretors only:** LNFPIII associated positively with total ASQ scores (standardized beta, 95% CI, *p*-value: β = 0.11; 95% CI, 0.03–0.19; *p* = 0.01), significant after Hochberg applied

^1^ Abbreviations: Ages and Stages Questionnaire, ASQ; Bayley Scales of Infant and Child Development, BSID-III; human milk oligosaccharides, HMOs; Kilifi Developmental Inventory, KDI; MacArthur-Bates Communicative Development Inventories, MB-CDIs; Mullen Scales of Early Learning, MSEL; fucosylated, Fuc; sialylated, Sia; 2′-fucosyllactose, 2′FL; 3-fucosyllactose, 3FL; 3′-sialyllactose, 3′SL; 6′-sialyllactose, 6′SL; lacto-N-tetraose, LNT; lacto-N-neotetraose, LNnT; lacto-N-fucopentaose; sialyl-LNT b, LSTb; sialyl-LNT c, LSTc; difucosyl-LNT, DFLNT; disialyl-LNT, DSLNT; lacto-N-hexaose, LNH; fucosyl-LNH, FLNH; difucosyl-LNH, DFLNH.

## Data Availability

Not applicable.
